# Humoral and cytokine responses to a heterologous goatpox vaccine in Mithun (*Bos frontalis*): a longitudinal field study

**DOI:** 10.3389/fimmu.2026.1818309

**Published:** 2026-04-21

**Authors:** Gundallahalli Bayyappa Manjunatha Reddy, Vikram Ramesh, Yallappa M. Somagond, Plabita Goswami, Selvaraj Ragulraj, Hlawndo Lalzampuia, Suchismitha Pal, Sudeep Nagaraj, Sunil Tadakod, Uzma Jabeen, Shraddha Bijalwan, Muthannan Andavar Ramakrishnan, Baldev Raj Gulati, Patil Shivanagowda Girish

**Affiliations:** 1 ICAR-National Institute of Veterinary Epidemiology and Disease Informatics, Bengaluru, Karnataka, India; 2 ICAR-National Research Centre on Mithun, Medziphema, Chumukedima, Nagaland, India; 3 ICAR – Indian Veterinary Research Institute, Bengaluru, Karnataka, India

**Keywords:** goatpox vaccine, lumpy skin disease, Mithun, northeast India, transboundary diseases

## Abstract

**Introduction:**

Lumpy Skin Disease (LSD), caused by a Capripoxvirus, has caused severe outbreaks among cattle in India. The disease status and vaccine response of Mithun (*Bos frontalis*), a semi-domesticated bovine species of major socio-economic importance in Northeast India, remain poorly understood. Because sero-epidemiological findings have indicated LSDV exposure in Mithun, preventive interventions are needed.

**Methods:**

We conducted a 12-month longitudinal field study in Nagaland to assess the safety and vaccine-induced immune response of a heterologous goatpox vaccine in seronegative Mithun. Humoral responses were monitored by indirect ELISA and virus neutralization test (VNT), whereas selected systemic cytokine markers (IFN-γ, IL-2, IL-4, and IL-10) were evaluated using serum IFN-γ ELISA and PBMC mRNA transcriptional profiling.

**Results:**

Spatial mapping supported continued LSDV activity in Mithun populations. The vaccine was safe, with no adverse clinical reactions or detectable viral shedding in sequential nasal swabs. Seroconversion and cytokine upregulation were evident after vaccination, with antibody responses and cytokine expression peaking at 30 days post-vaccination.

**Discussions:**

These findings provide field-based evidence of vaccine-induced humoral and cytokine responses to the heterologous goatpox vaccine in Mithun and generate baseline information for future evaluation of LSD control strategies in this species. Antigen-specific cell-mediated immunity (CMI) was not assessed in this study, as stimulated PBMC recall assays were beyond the scope of the present field study, and should be evaluated in future investigations.

## 1 Introduction

Mithun (*Bos frontalis*), a native semi-domesticated bovine species, is found naturally in the thick forests and mountainous regions of Northeast India, especially in Arunachal Pradesh, Nagaland, Manipur, and Mizoram. Locally referred to as ‘gayal’, Mithun is of great socio-economic, ecological, and cultural importance to the tribal communities in these areas (1). While it has traditionally been raised for meat, Mithun also plays a crucial role in traditional exchanges and social rituals. However, due to its limited population and high ecological sensitivity, the International Union for Conservation of Nature (IUCN) has classified Mithun as a ‘vulnerable’ species (1).

Lumpy Skin Disease (LSD) is a highly impactful, fast-spreading transboundary disease caused by the Lumpy Skin Disease Virus (LSDV), a double-stranded DNA virus from the Capripoxvirus genus (Poxviridae) (2). The disease is characterized by symptoms such as fever, numerous firm skin nodules, lymph node enlargement, severe weight loss, and reproductive issues, leading to devastating economic losses in the livestock industry (3). The virus is mainly spread mechanically by blood-feeding arthropods, although direct and indirect transmission routes also contribute to its spread (4). Due to its high morbidity and rapid spread, the World Organization for Animal Health (WOAH) classified LSD as a globally notifiable disease (5).

LSD was first confirmed in India in 2019 (6). The subsequent large-scale outbreaks affected large scale cattle population across India, causing significant mortalities and severely impacting milk production systems with an estimated economic loss of USD 2440.29 million (90% CI 2162.55–2716.15)/(INR 202,544.07 million (90% 179,491.65—2,225,440.45) during 2022 & 2023 (7). While cattle (Bos taurus/indicus) and water buffalo are known primary hosts, new evidence indicates that related bovine species, such as yak and Mithun, are highly susceptible to LSDV infections (8, 9). Despite this, the immunological susceptibility of Mithun to LSDV and the effectiveness of existing vaccines remains largely unexplored (5).

An extensive sero-epidemiological investigation of LSD in Mithun was conducted across Nagaland and Arunachal Pradesh, demonstrating natural exposure in these regions. Given the limited evidence for the use of existing cattle vaccines in Mithun, we undertook a longitudinal field study to evaluate the safety and vaccine-induced immune response of the heterologous goatpox vaccine over a 12-month period. To our knowledge, this is the first field-based assessment of heterologous goatpox vaccination in Mithun and provides baseline information relevant to disease monitoring and future vaccination studies in this vulnerable species.

## 2 Materials and methods

### 2.1 Cells, virus and animals

The Madin Darby Bovine Kidney (MDBK clone 118; NCCS) cell line was cultured in Minimum Essential Medium (MEM) (HiMedia, India) supplemented with 10% fetal bovine serum, 100 IU/mL penicillin (Sigma), 100 μg/mL streptomycin (Sigma), and 100 μg/mL kanamycin (Sigma) at 37 °C in a 5% CO2 atmosphere. For the virus neutralization test (VNT), the LSDV/Cattle/India/Chitradurga/P34 strain (GenBank accession OR863389), isolated by ICAR-NIVEDI and propagated in MDBK cells, was used.

In this longitudinal field study, 26 Mithun were selected after screening for seronegativity by both indirect ELISA and VNT. Vaccinated animals were maintained under routine field conditions at the experimental facility at ICAR-National Research Centre on Mithun, Medziphema, Nagaland. An unvaccinated seronegative group was maintained at a geographically separate location under similar management conditions to serve as the negative control group. This geographic separation was intended to minimize potential exposure of control animals to vaccine virus or natural infection during the observation period.

The intervention involved a commercially available live-attenuated freeze-dried goatpox virus vaccine (Uttarkashi strain). A standard dose of 1 mL (containing 10³ TCID_50_) was administered subcutaneously by designated field veterinarians, following the guidelines of the Department of Animal Husbandry and Dairying (DAHD), Government of India.

After vaccination, the animals were monitored clinically each day for 30 days. Observations focused on ocular and nasal discharge, fever, local reactions at the injection site, and the appearance of characteristic nodular lesions. Sequential nasal washes were collected on days 0, 9, 15, 23, 30, 300, and 365 for PCR-based assessment of possible LSDV shedding.

### 2.2 Ethics statement

Approval for collection of blood and nasal wash samples was obtained from the Animal Husbandry and Veterinary Services Department, Government of Nagaland. All procedures followed institutional animal welfare guidelines and were performed in accordance with ARRIVE 2.0 recommendations. The blood was subjected for routine hematological parameters before and after vaccination.

### 2.3 Spatial epidemiology and seroprevalence

The macro-spatial distribution of Mithun populations throughout the Northeastern Region (NER), along with the district-specific patterns of LSD incidence in Nagaland and Arunachal Pradesh, were mapped using the Quantum Geographic Information System (QGIS, version 3.32.2). The primary demographic data was sourced from the 20^th^ Livestock Census conducted by the Government of India.

### 2.4 Indirect enzyme-linked immuno-sorbent assay

Humoral responses were measured using an indirect ELISA based on recombinant capripoxvirus antigen validated at ICAR-NIVEDI for detection of capripoxvirus-specific antibodies. The recombinant antigen corresponded to the A27L gene of the field isolate LSDV/CHITRA-05/NIVEDI/ICAR/2020/India (GenBank accession OR863389) using a prokaryotic expression system. ELISA plates were coated with purified antigen at 50 ng/well and blocked with 1% gelatin. Test sera were diluted 1:150 and incubated with rabbit anti-bovine IgG-HRP conjugate at 1:7500 dilution. Color was developed with OPD, the reaction was stopped with 1 M H_2_SO_4_, and optical density was measured at 492 nm. Percentage positivity (PPV) values greater than 35% were considered positive.

### 2.5 Virus neutralization test

The VNT was performed as described previously (9). Briefly, sera were heat-inactivated at 56 °C for 30 min and subjected to serial two-fold dilutions starting at 1:4. Diluted sera were incubated for 1 h at 37°C with 100 TCID_50_ of LSDV/Cattle/India/Chitradurga/P34 and then inoculated onto MDBK cell monolayers seeded at 2 × 10^4^ cells/well in 96-well plates. Cytopathic effect (CPE) was monitored for 6 days. Neutralizing titres of ≥1:8 were interpreted as evidence of seroconversion, and endpoint values were expressed logarithmically (log_10_).

### 2.6 Interferon gamma assay

Systemic IFN-γ levels were quantified as a supportive cytokine marker using a commercial Bovine Interferon-γ ELISA Kit from BT LAB, China, following the instructions provided by the manufacturer. Serum samples were incubated with designated Bovine IFN-γ capture antibodies and a secondary streptavidin-HRP conjugate. After the reaction was stopped, optical density values were accurately measured at 450 nm using a spectrophotometer (Tecan Infinite F50, Switzerland). It should be note that this serum-based IFN-γ measurement does not constitute direct evidence of antigen-specific cell-mediated immunity (CMI), which would require stimulated PBMC recall assays.

### 2.7 Molecular confirmation of LSDV exclusion

To exclude possible subclinical LSDV infection, total DNA was extracted from sequential nasal swabs using the DNeasy Blood & Tissue Kit (Qiagen). Samples underwent three freeze-thaw cycles and vortexing in 200 µL viral transport medium. DNA templates were amplified in 25 µL reactions using P32 gene primers (Forward: 5′-TCCGAGCTCTTTCCTGATTTTTCTTACTAT-3′; Reverse: 5′-TATGGTACCTAAATTATATACGTAAATAAC-3′) 8 with 2X DreamTaq Green PCR Master Mix (Thermo, USA). Thermal cycling comprised an initial denaturation at 94 °C for 5 min, followed by 30 cycles of 94 °C for 1 min, 52 °C for 30 s, and 72 °C for 1 min, with a final extension at 72 °C for 5 min. Amplicons were visualized on 1.5% agarose gel electrophoresis (**Table 1**).

**Table 1:** PCR protocol performed for LSDV detection in samples.

Stage	Step	Temperature (°C)	Time
Initial denaturation	1	94	5 min
30 cycles	Denaturation	94	1 min
	Annealing	52	30 sec
	Extension	72	1 min
Final extension	1	72	5 min

### 2.8 Cytokine gene expression profiling

Peripheral Blood Mononuclear Cells (PBMCs) were effectively separated from fresh whole blood, which was collected in EDTA vacutainers, using Histopaque density gradient centrifugation (Sigma Aldrich) within 8 hours after collection. RNA was extracted from the isolated PBMCs with the RNeasy Plus Kit (Qiagen), and this was immediately followed by the synthesis of high-quality cDNA using the RevertAid First Strand Synthesis system (Thermo Fisher).

We monitored the specific relative transcriptional expression of typical Th1 (IL-2, IFN-γ) and Th2 (IL-4, IL-10) cytokines through the QuantStudio 3 Real-Time PCR platform (Applied Biosystems). The specific oligonucleotide primers for these genes are listed in **Table 2** as the thermal profile in **Table 3**. GAPDH served as the consistently stable endogenous reference. The relative fold-changes were calculated using the comparative threshold cycle method (R = 2-ΔΔCT) as described by Livak and Schmittgen (12).

**Table 2:** The details of primer sequences used cytokines studies in real-time PCR.

Gene	Primer sequence (5’–3’)	Tm (°C)	Size (bp)	Accession No.	Reference
IL-2	F:TTTTACGTGCCCAAGGTTTAA R:CGTTTACTGTTGCATCATCA	52	217	M12791	(10)
IL-4	F:CAAAGAACACAACTGAGAAG R:AGGTCTTTCAGCGTACTTGT	55	181	M77120	
IL-10	F:TGCTGGATGACTTTAAGGG R:AGGGCAGAAAGCGATGACA	55	186	U00799	
IFN-γ	F:ATAACCAGGTCATTCAAAGG R:ATTCTGACTTCTCTTCCGCT	55	218	M29867	
GAPDH	F: GCGTGAACCACGAGAAGTATAA R:CCCTCCACGATGCCAAAGT	59	194	—	(11)

**Table 3:** Protocol in the thermal cycler for real-time PCR.

Segment	Cycle(s)	Temperature (°C)	Duration
1	1	50	2 min
2	1	95	10 min
3	40	Respective Tm	1 min
4	1	60	1 min

### 2.9 Statistical analysis

Statistical analyses, including Tukey’s *post hoc* test and kappa index assessment, were performed using GraphPad Prism (v8.0.1) and IBM SPSS (v27). A p value of <0.05 was considered statistically significant.

## 3 Results

### 3.1 Epidemiology and seroprevalence of LSD in Mithun

**Figure 1:**
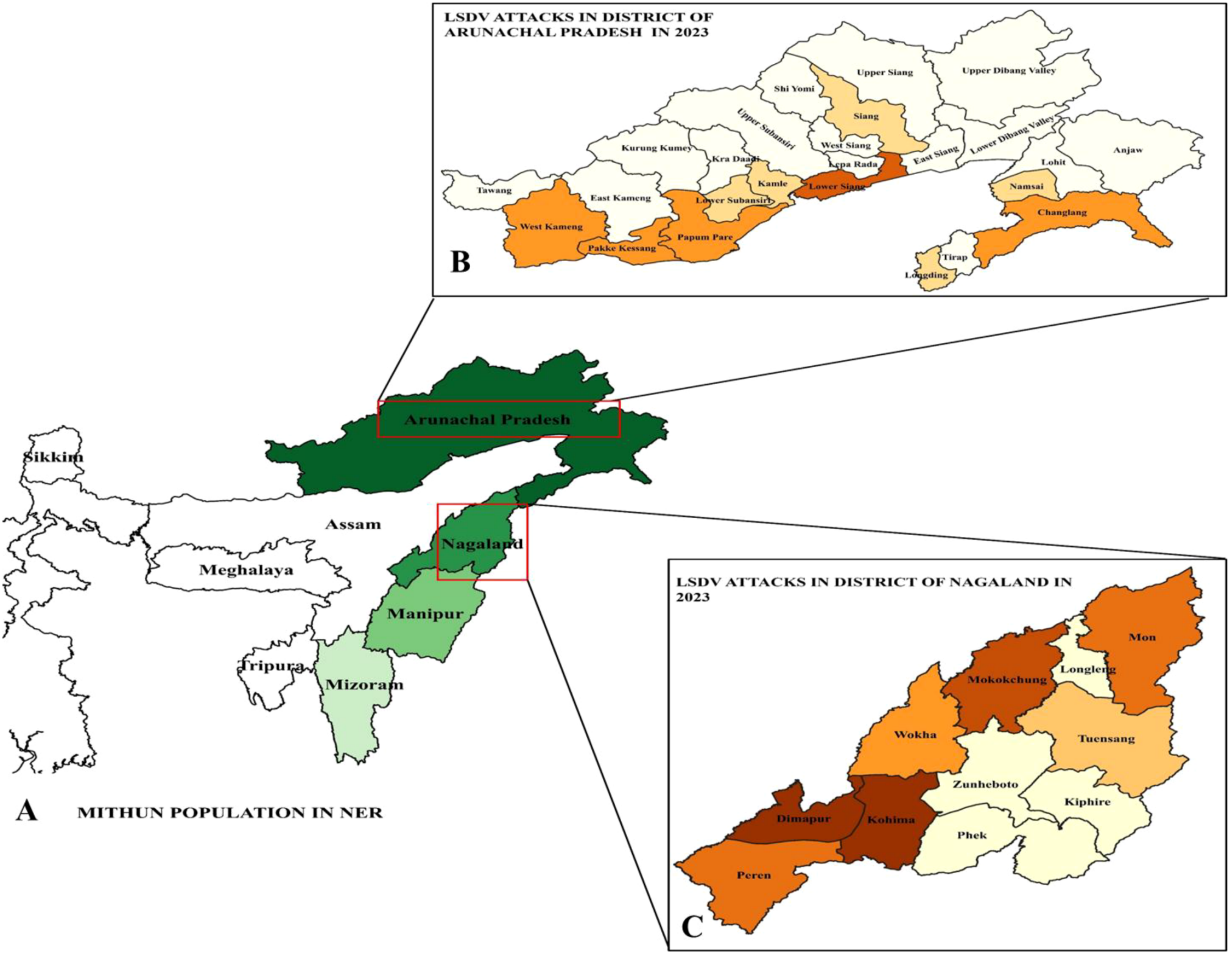
Study on Mithun population and LSD seroprevalence. **(A)** Map illustrating the Mithun population in Nagaland and Arunachal Pradesh, with color intensity indicating population density. The number of LSD attacks reported during the study period in Arunachal Pradesh **(B)** and Nagaland **(C)**.

Spatial analysis showed that Mithun populations are concentrated mainly in Arunachal Pradesh and Nagaland (**Figure 1**). Demographic risk-factor distributions according to sex, geography, and age are presented in **Supplementary Figures 1A, B**. Comparison of census data from 1997 to 2019 indicated a marked increase in population in Arunachal Pradesh, whereas Nagaland showed a slight decline (**Supplementary Figure 1C**). Seroprevalence mapping further indicated natural exposure to LSDV in semi-domesticated Mithun herds (**Supplementary Figure 1D**).

### 3.2 Clinical safety of goatpox vaccination

Throughout the observation period, Mithun showed good tolerance to the heterologous goatpox vaccine. There was no evidence of local swelling at the injection site, and clinical parameters such as rectal temperature and feed consumption remained within normal ranges. A clinical assessment conducted within the first 30 days after vaccination showed no signs of nodular lesions or fever. The blood parameters did not vary before and after vaccination (**Supplementary Figure 2**). Additionally, repeated PCR tests on nasal swabs were negative for capripoxvirus DNA, suggesting no detectable viral shedding occurred during the monitoring period.

### 3.3 Longevity of humoral responses

**Figure 2:**
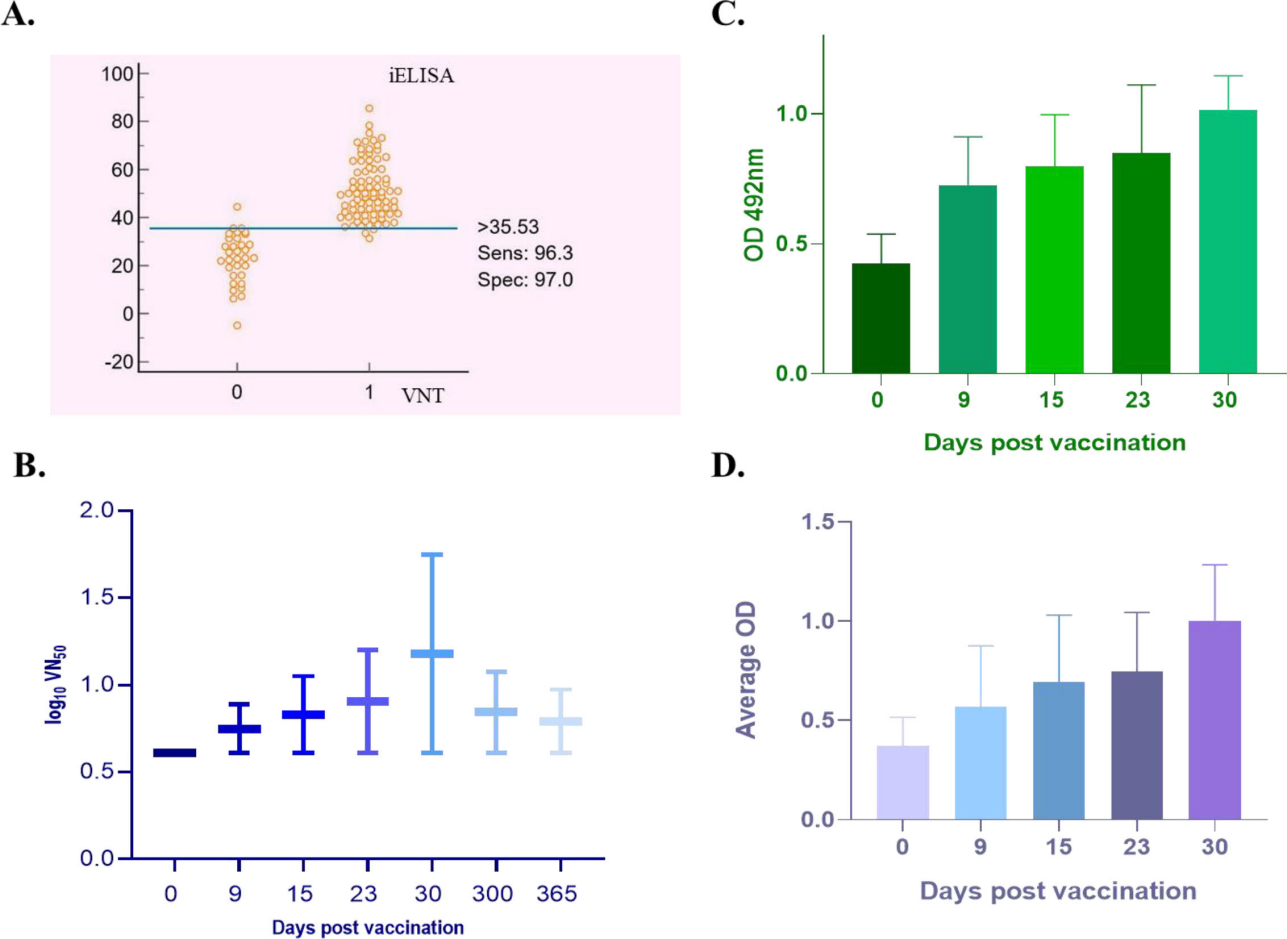
Immune response of the goatpox vaccine in Mithun. **(A)** Dot plot depicting the kappa value which assesses the agreement between VNT and indirect ELISA. **(B)** Graph depicting VNT titres in log_10_VN_50_ values in vaccinated animals. **(C)** Bar graph depicting the indirect ELISA data as OD values read at 492nm from serum samples collected at various time points post vaccination. **(D)** Bar graph depicting the IFN-γ concentration from PBMC samples collected at various time points post vaccination.

Humoral responses were monitored by indirect ELISA and VNT using samples collected at 0, 9, 15, 23, 30, 300, and 365 days post-vaccination (dpv). Among 170 sequential serum samples, 141 and 144 were positive by indirect ELISA and VNT, respectively. The kappa index was 0.899, indicating almost perfect agreement between the two assays (**Figure 2A**, **Supplementary Figure 3**).

VNT showed a progressive rise in neutralizing antibody titres, with a peak at 30 dpv followed by gradual decline up to 365 dpv (**Figure 2B**). Indirect ELISA showed rapid seroconversion, with 68% positivity at day 9, 91.3% at day 15, and complete seropositivity by day 30 (**Figure 2C**).

Systemic immune activation measured by IFN-γ ELISA was detectable by day 9 and increased progressively, reaching a peak at 30 dpv in parallel with the humoral response (**Figure 2D**).

### 3.4 Cytokine expression pathways and Th1/Th2 balance

**Figure 3:**
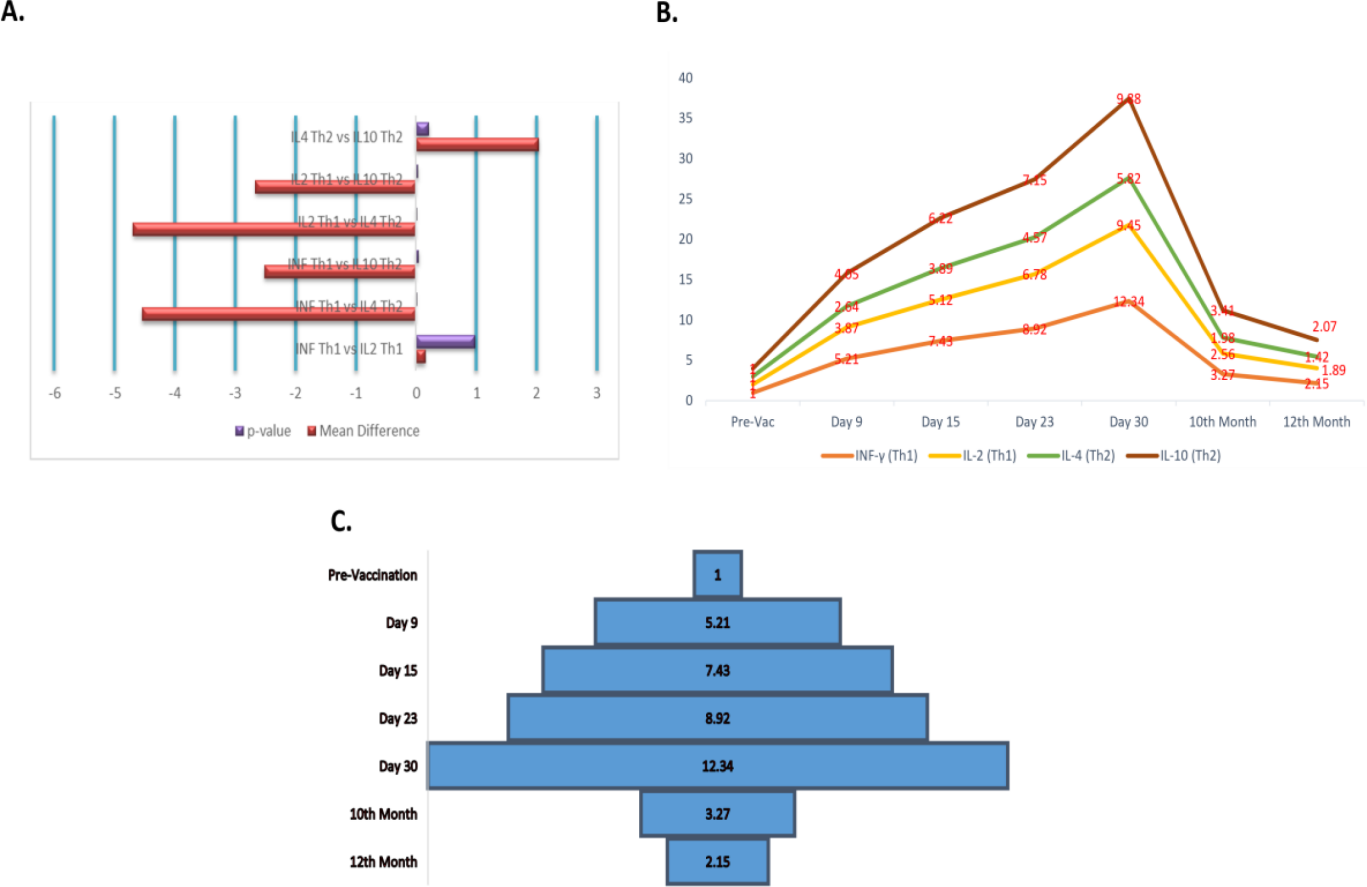
**(A)** Bar chart comparing cytokines associated with Th1 and Th2 responses. **(B)** Line chart showing cytokine expression profiles over the period after vaccination. **(C)** Graph showing fold changes in cytokine levels at different time points after vaccination.

Analysis of cytokine mRNA expression showed clear immunological changes after vaccination. Within the Th1-associated cytokines, IFN-γ and IL-2 did not differ significantly (p = 0.982; mean difference = 0.150). In contrast, IFN-γ expression was significantly different from IL-4 (p = 0.001; mean difference = -4.546) and IL-10 (p = 0.042; mean difference = -2.511). IL-2 expression also differed significantly from IL-4 (p = 0.001) and IL-10 (p = 0.032). No significant difference was observed between the Th2-associated cytokines IL-4 and IL-10 (p = 0.210) (**Figure 3A**, **Table 4**).

**Table 4:** Comparing ΔCt values between cytokine groups in vaccinated Mithun.

Comparison	Mean difference	p-value	Significance
IFN-γ (Th1) vs IL-2 (Th1)	0.150	0.982	NS
IFN-γ (Th1) vs IL-4 (Th2)	-4.546	0.001	Significant
IFN-γ (Th1) vs IL-10 (Th2)	-2.511	0.042	Significant
IL-2 (Th1) vs IL-4 (Th2)	-4.696	0.001	Significant
IL-2 (Th1) vs IL-10 (Th2)	-2.661	0.032	Significant
IL-4 (Th2) vs IL-10 (Th2)	2.035	0.210	NS

### 3.5 Temporal dynamics of cytokine activation

Before vaccination, transcript levels of IFN-γ, IL-2, IL-4, and IL-10 were set at 1.00 ± 0.00. By 9 dpv, early immune activation was evident, with fold increases of 5.21 for IFN-γ, 3.87 for IL-2, 2.64 for IL-4, and 4.05 for IL-10. Expression continued to increase through 15 and 23 DPV and reached a peak at 30 dpv. At this time point, IFN-γ reached 12.34 ± 0.85 fold, followed by IL-10 (9.88 ± 0.81), IL-2 (9.45 ± 0.73), and IL-4 (5.82 ± 0.42), indicating peak activation of both humoral and cytokine responses.

After 30 dpv, cytokine expression declined gradually. By month 10, IFN-γ (3.27 ± 0.24) and IL-10 (3.41 ± 0.29) approached a plateau. At 12 months, all markers moved closer to baseline values: IFN-γ (2.15 ± 0.18), IL-2 (1.89 ± 0.16), IL-4 (1.42 ± 0.12), and IL-10 (2.07 ± 0.20) (**Figures 3B, C**).

## 4 Discussion

Our earlier epidemiological work indicated that LSD outbreaks in Mithun herds were associated with transmission from adjacent cattle populations. Although heterologous goatpox vaccine has been used for LSD control in cattle, information on vaccine response in Mithun has been lacking. The present study therefore evaluated vaccine-induced humoral and cytokine responses and field safety of the goatpox vaccine in Mithun over a 12-month period. Within the limits of the study design, the results show that vaccination was associated with measurable humoral and cytokine responses in this species.

It is important to contextualize this study within current national vaccination policy. While there have been significant developments regarding homologous LSD vaccines, recent official data confirm the ongoing operational mandate for heterologous platforms. Specifically, the Standing Committee on Agriculture, Animal Husbandry and Food Processing (2023-24) has reiterated a national vaccination strategy that communicates an indicative timeline (April to June) for annual vaccination and re-vaccination of all eligible cattle using the available goatpox vaccine-Uttarkashi Strain [Available at: https://eparlib.sansad.in]. In this context, the present study on Mithun (*Bos frontalis)* provides critical baseline profiles of humoral and cytokine responses to the goatpox vaccine, filling a significant knowledge gap and supporting the evidence-based implementation of current national vaccination strategies for this vulnerable endemic species. Furthermore, following the commercialization of homologous live-attenuated LSD vaccines (DAHD, June 2025) and the subsequent guidelines (DAHD, August 2025), the latest DAHD advisory (March 02, 2026) recommends transitioning to the homologous vaccine while explicitly mandating a one-year gap from the date of previous goatpox vaccination. In light of this, understanding the duration of the immunological response to the goatpox vaccine as evaluated in the present study remains critical for maintaining herd immunity until the homologous vaccine can be safely administered in Mithun species.

The strong agreement between indirect ELISA and VNT (kappa = 0.899) indicates consistency between the two serological approaches and is comparable to previously reported cattle assays (13). Both assays showed seroconversion after vaccination, with the highest responses observed around 30 dpv. Because no challenge study was performed, these findings should be interpreted as evidence of vaccine-induced immune response rather than direct proof of protection. Nevertheless, the combined serological and cytokine data provide a useful first immunological profile of goatpox vaccination in Mithun under field conditions.

The increase in IFN-γ detected by ELISA and the transcriptional changes observed in IFN-γ, IL-2, IL-4, and IL-10 indicate systemic immune response after vaccination. Similar early IFN-γ responses have been described in cattle and other capripoxvirus studies (14–17). In the present work, these measurements were interpreted as supportive indicators of vaccine-associated immune responses rather than as definitive evidence of antigen-specific recall responses. This distinction is important because antigen-specific stimulation assays were beyond the scope of the present study.

IL-10 is an important immunoregulatory cytokine that can modulate Th1-associated inflammation while supporting humoral immune responses (18–21). The concurrent increase in IL-4 and IL-10 observed here is consistent with activation of pathways associated with antibody production and immune regulation. Similar cytokine trends have been reported in cattle with LSDV-associated immune responses (10).

At the same time, the increases in IFN-γ and IL-2 are consistent with activation of Th1-associated pathways that contribute to antiviral cellular responses. Together, these observations suggest a mixed but coordinated immune response after vaccination, with early activation followed by gradual decline over time. This pattern is in line with the broader immunogenicity profile expected after capripoxvirus vaccination (5, 11, 22).

Consequently, the serum IFN-γ ELISA and PBMC cytokine data presented in this study should be interpreted strictly as systemic cytokine markers indicative of vaccine-associated immune activation not as definitive evidence of antigen-specific cell-mediated immunity. Future investigations should incorporate antigen-specific recall assays to comprehensively characterise CMI responses in Mithun herd.

Overall, the heterologous goatpox vaccine elicited measurable humoral and cytokine responses in Mithun and was tolerated under the conditions of this study. These data provide baseline information for future work evaluating larger cohorts, antigen-specific cellular responses, homologous LSD vaccines, and direct protection studies in Mithun.

### 4.1 Study limitations

This study has some limitations like antigen-specific cellular immune responses were not evaluated using recall assays such as PBMC stimulation or ELISPOT. The work was conducted under field conditions in Mithun, a species for which immunological reagents and standardized assays remain limited. A long-term parallel unvaccinated cohort was not maintained throughout the full study period because the primary objective was to assess vaccine-induced immunogenicity in seronegative animals after vaccination. In addition, a controlled challenge study was beyond the scope of the present work. Hence, the findings should be interpreted in the context of the field setting and study period. The study was designed to characterize vaccine-induced immune responses in Mithun under operational conditions, not to compare different vaccine platforms or to establish national vaccination policy. Accordingly, the conclusions of the study are restricted to field-based immunogenicity observations in this species.

## 5 Conclusions

The present study shows that heterologous goatpox vaccination in Mithun was associated with measurable antibody responses, cytokine modulation, and good clinical tolerance under field conditions. The strongest responses were observed around 30 days after vaccination, followed by gradual decline over the 12-month observation period. These findings provide field-based evidence of vaccine-induced humoral and cytokine responses in Mithun; however, further studies incorporating larger populations, antigen-specific cellular assays, and challenge or comparative vaccine evaluations are needed before drawing conclusions about protective efficacy. Antigen-specific cell-mediated immunity was not assessed and should be a priority for future investigations using recall assays.

## Data Availability

The original contributions presented in the study are included in the article/**Supplementary Material**. Further inquiries can be directed to the corresponding authors.

## Supplementary material

Supplementary Figure 1 Panels showing distributions of Mithun risk factors, namely sex, area, and age, in Nagaland **(A)** and Arunachal Pradesh **(B)**. Graph showing year-wise Mithun population dynamics in both states **(C)** and a Pie chart showing LSD seroprevalence in Mithun populations in the study states **(D)**.

Supplementary Figure 2 Bar diagram representing the different blood parameters studied before and after vaccination with goatpox vaccine in Mithun. There were no significant variations (ns) recorded during the study.

Supplementary Figure 3 ROC analysis between ELISA and serum neutralization test generated using MedCalc software.

## References

[1] Dorji T Wangdi J Shaoliang Y Chettri N Wangchuk K . Mithun (Bos frontalis): the neglected cattle species and their significance to ethnic communities in the Eastern Himalaya—a review. Anim Bioscience. (2021) 34:1727. doi: 10.3329/bjvm.v12i2.21277. PMID: 33902178 PMC8563247

[2] Khan YR Ali A Hussain K Ijaz M Rabbani AH Khan RL . A review: Surveillance of lumpy skin disease (LSD), a growing problem in Asia. Microb Pathogen. (2021) 158:105050. doi: 10.1016/j.micpath.2021.105050. PMID: 34146642

[3] Kumar N Sharma S Tripathi BN . Pathogenicity and virulence of lumpy skin disease virus: a comprehensive update. Virulence. (2025) 16:2495108. doi: 10.1080/21505594.2025.2495108. PMID: 40265421 PMC12036493

[4] Bayyappa MRG Pabbineedi SM Nagaraj S Bijalwan S Tadakod S Uma CR . Spatiotemporal epidemiology of lumpy skin disease and evaluation of the heterologous goatpox vaccine: insights into immunogenicity and impact. Vaccines. (2025) 13:641. doi: 10.3390/vaccines13060641. PMID: 40573972 PMC12197790

[5] Reddy GBM Mounica PS Sudeep N Vikram R Garam GB Lalzampuia H . First evidence of lumpy skin disease in mithun (Bos frontalis) in India. Arch Virol. (2024) 169:65. doi: 10.1007/s00705-024-05996-7. PMID: 38451344

[6] Sudhakar SB Mishra N Kalaiyarasu S Jhade SK Hemadri D Sood R . Lumpy skin disease (LSD) outbreaks in cattle in Odisha state, India in August 2019: epidemiological features and molecular studies. Transboundary Emerging Dis. (2020) 67:2408–22. doi: 10.1111/tbed.13579. PMID: 32304275

[7] Naidu GG Shivappa RR Rajanna PR Gondali H Devaraju MH Nagesh P . Assessment of economic burden of lumpy skin disease in India using stochastic modeling. Sci Rep. (2025) 15:10160. doi: 10.1038/s41598-025-94383-6. PMID: 40128344 PMC11933382

[8] Manjunathareddy GB Saminathan M Sanjeevakumar L Rao S Dinesh M Dhama K . Pathological, immunological and molecular epidemiological analysis of lumpy skin disease virus in Indian cattle during a high-mortality epidemic. Vet Q. (2024) 44:1–22. doi: 10.1080/01652176.2024.2398211. PMID: 39233648 PMC11378666

[9] Manjunatha Reddy GB Pabbineedi SM Nagaraj S Bijalwan S Tadakod S Bhutia Z . Lumpy skin disease (LSD) in yak (Bos grunniens): an evidence of species spillover from cattle in India. Microorganisms. (2023) 11:2823. doi: 10.3390/microorganisms11122823. PMID: 38137967 PMC10746030

[10] Memon MA Abro SH Kalhoro DH Kalhoro NH Abro R . Immune response in cattle following immunization with a heterologous lumpy skin disease virus vaccine: a longitudinal study. Pak-Euro J Med Life Sci. (2024) 7:S345–52.

[11] Konnai S Tajima S Tajiki M Goto Y Mamba K Ohashi S . Rapid quantitative analysis of bovine cytokine genes by real-time RT-PCR. Veterinary Microbiol. (2003) 94:283–94. doi: 10.1016/s0378-1135(03)00119-6. PMID: 12829382

[12] Livak KJ Schmittgen TD . Analysis of relative gene expression data using real-time quantitative PCR and the 2(-Delta Delta C(T)) method. Methods. (2001) 25:402–8. doi: 10.1006/meth.2001.1262. PMID: 11846609

[13] Samojlović M Polaček V Gurjanov V Lupulović D Lazić G Petrović T . Detection of antibodies against lumpy skin disease virus by virus neutralization test and ELISA methods. Front Vet Sci. (2019) 6:470. 31998759

[14] Norian R Afzal Ahangran N Varshovi HR Azadmehr A . Evaluation of cell-mediated immune response in PBMCs of calves vaccinated by capri pox vaccines using ELISA and real-time RT-PCR. Res Mol Med. (2017) 5:3–8. doi: 10.29252/rmm.5.2.3. PMID: 36812099

[15] Kresic N Philips W Haegeman A de Regge N . Evaluation of an interferon-gamma release assay for early detection of lumpy skin disease virus infection and vaccination in cattle. Microbiol Spectr. (2025) 13:e02939-24. doi: 10.1128/spectrum.02939-24. PMID: 40062882 PMC11960450

[16] Chibssa TR Kangethe RT Berguido FJ Settypalli TBK Liu Y Grabherr R . Innate immune responses to wildtype and attenuated sheeppox virus mediated through RIG-1 sensing in PBMC in-vitro. Front Immunol. (2021) 12:666543. doi: 10.3389/fimmu.2021.666543. PMID: 34211465 PMC8240667

[17] Couper KN Blount DG Riley EM . IL-10: the master regulator of immunity to infection. J Immunol. (2008) 180:5771–7. doi: 10.4049/jimmunol.180.9.5771. PMID: 18424693

[18] Raphael I Nalawade S Eagar TN Forsthuber TG . T cell subsets and their signature cytokines in autoimmune and inflammatory diseases. Cytokine. (2015) 74:5–17. doi: 10.1016/j.cyto.2014.09.011. PMID: 25458968 PMC4416069

[19] Ahmad SF Patra MK Mahendran K Paul BR Khanna S Singh AK . Hematological and serum biochemical parameters and profiling of cytokine genes in lumpy skin disease in Vrindavani cattle. 3 Biotech. (2023) 13:66. doi: 10.1007/s13205-023-03477-3. PMID: 36721645 PMC9884329

[20] Bayyappa MRG Uma CR Bijalwan S Tadakod S Nagaraj S Naragund M . Molecular epidemiological and spatiotemporal analysis of lumpy skin disease outbreaks in cattle from Karnataka, India. Front Cell Infect Microbiol. (2025) 15:1596973. doi: 10.3389/fcimb.2025.1596973. PMID: 40630638 PMC12234529

[21] Zaros LG Bricarello PA Amarante AFT Coutinho LL . Quantification of bovine cytokine gene expression using real-time RT-PCR methodology. Genet Mol Biol. (2007) 30:575–9. doi: 10.1590/s1415-47572007000400012. PMID: 41880456

[22] Boshra H Truong T Nfon C Bowden TR Gerdts V Tikoo S . A lumpy skin disease virus deficient of an IL-10 gene homologue provides protective immunity against virulent capripoxvirus challenge in sheep and goats. Antiviral Res. (2015) 123:39–49. doi: 10.1016/j.antiviral.2015.08.016. PMID: 26341190

