# Supplementary material for: Humoral and cytokine responses to a heterologous goatpox vaccine in Mithun (Bos frontalis): a longitudinal field study
Source: Front Immunol. 2026 Apr 21;17:1818309. doi: 10.3389/fimmu.2026.1818309 (PMC13138996; doi:10.3389/fimmu.2026.1818309)
Supplement: Supplementary Figure 1 — Panels showing distributions of Mithun risk factors, namely sex, area, and age, in Nagaland (A) and Arunachal Pradesh (B). Graph showing year-wise Mithun population dynamics in both states (C) and a Pie chart showing LSD seroprevalence in Mithun populations in the study states (D). [file DataSheet1.docx]

**Supplementary figures**

**
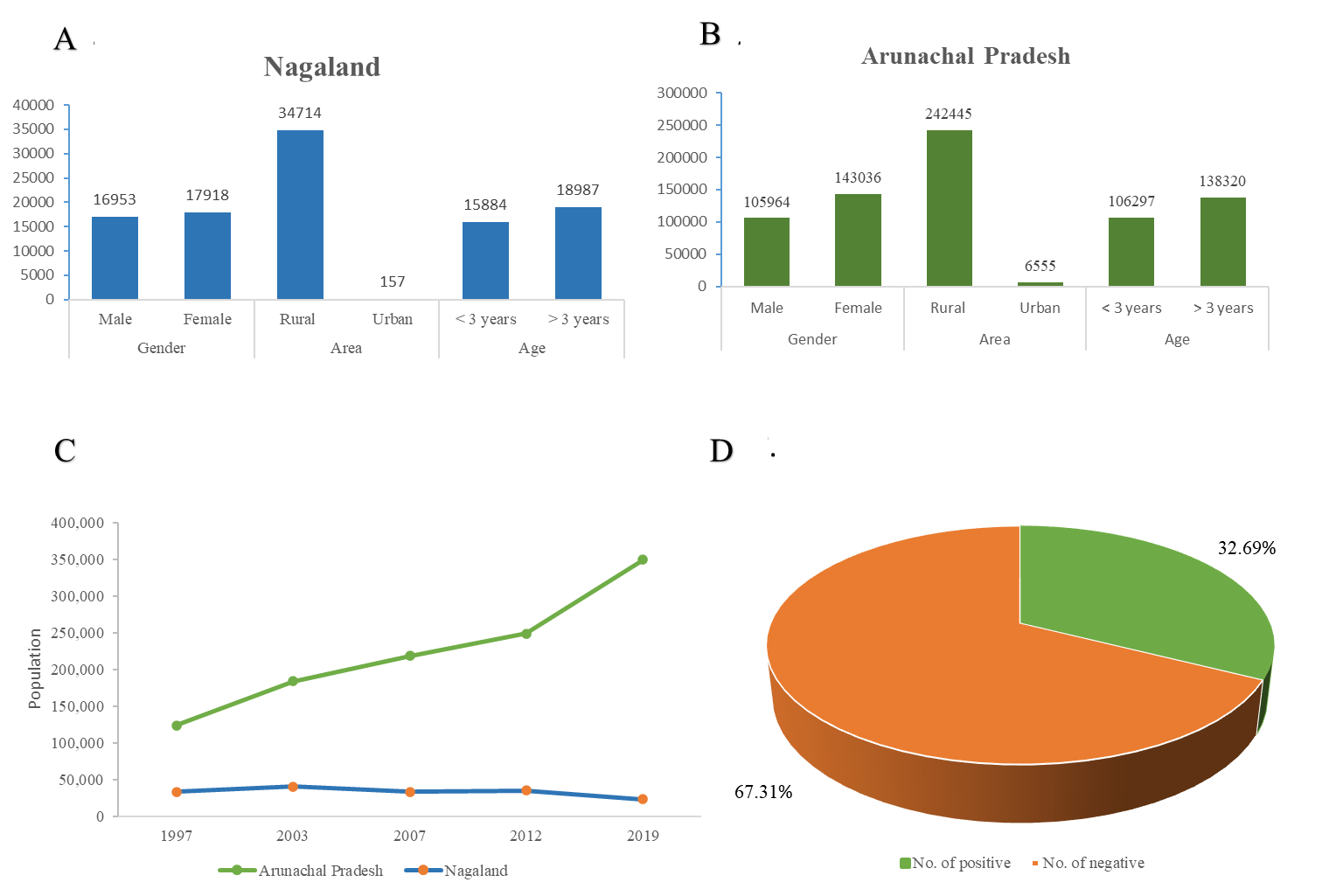
**

Supplementary Figure 1. Panels showing distributions of Mithun risk factors, namely sex, area, and age, in Nagaland (A) and Arunachal Pradesh (B). Graph showing year-wise Mithun population dynamics in both states (C) and a Pie chart showing LSD seroprevalence in Mithun populations in the study states (D).


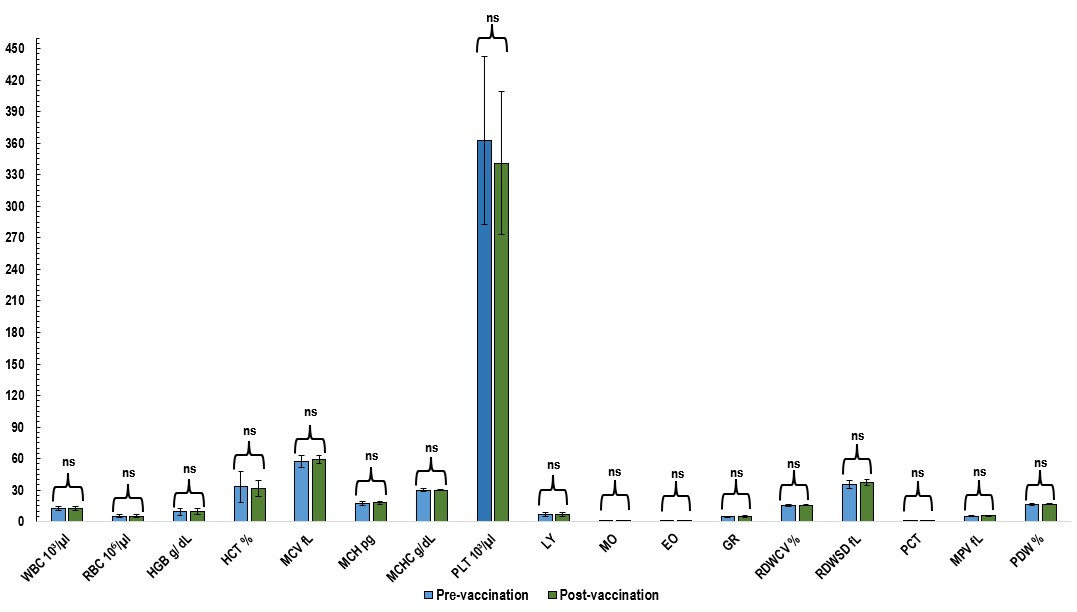


Supplementary Figure 2. Bar diagram representing the different blood parameters studied before and after vaccination with goatpox vaccine in Mithun. There were no significant variations (ns) recorded during the study,


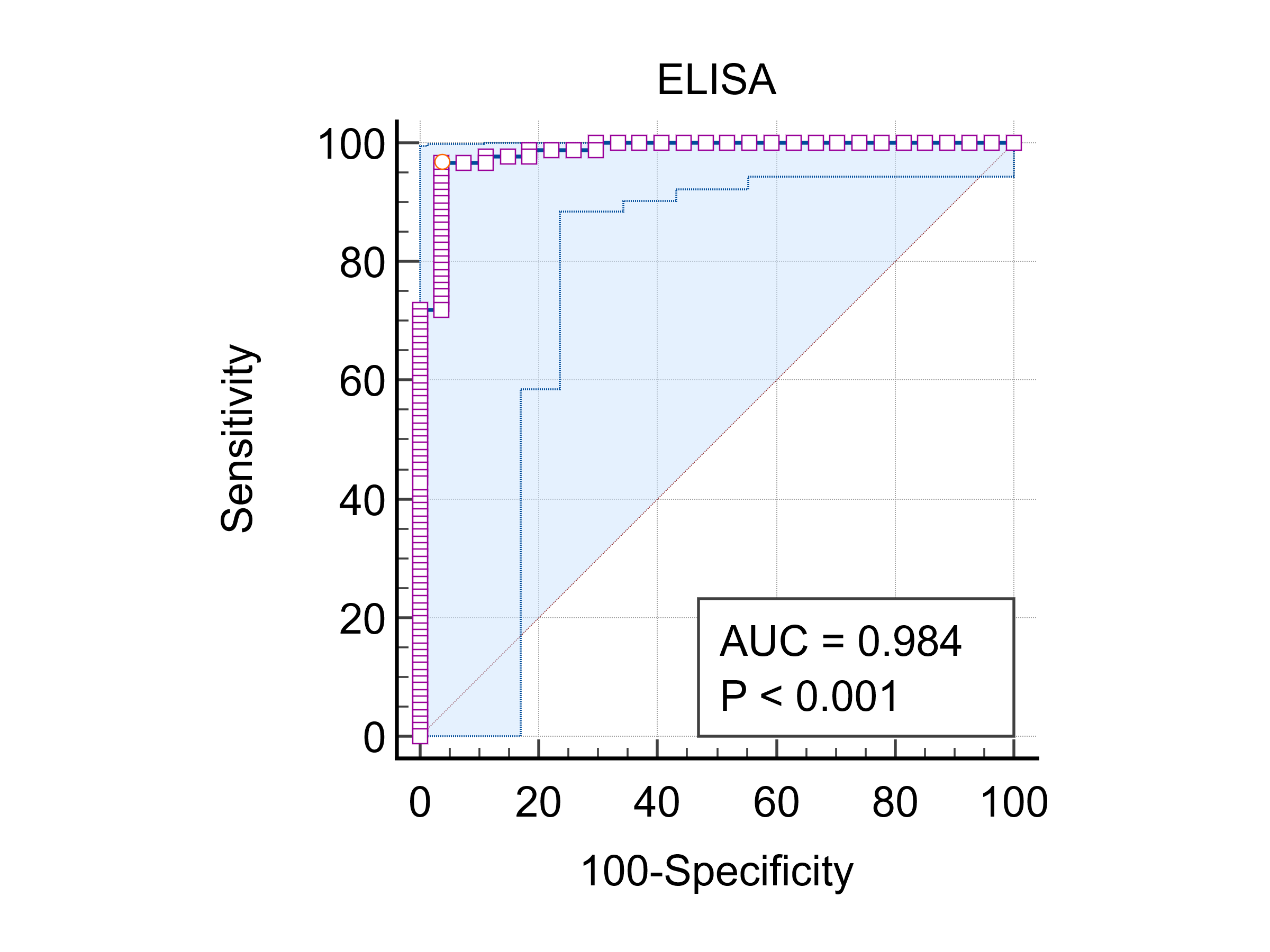


Supplementary Figure 3. ROC analysis between ELISA and serum neutralization test generated using MedCalc software.
